# Functional Neurological Disorder: a multidisciplinary approach

**DOI:** 10.1192/j.eurpsy.2022.1002

**Published:** 2022-09-01

**Authors:** C. Alvarez Garcia, A. Gomez Martín, M.D.C. Molina Liétor, I. Cuevas Iñiguez, A. Sanz Giancola

**Affiliations:** 1 Hospital Universitario Príncipe de Asturias, Psychiatry, Alcalá de Henares, Spain; 2 Hospital Universitario Príncipe de Asturias, Psiquiatría, Alcalá de Henares, Spain; 3 Hospital Principe de Asturias, Psiquiatría, Alcala de Henares, Spain

**Keywords:** multidisciplinary, neurological, conversion

## Abstract

**Introduction:**

Functional neurological disorders (FNDs), also known as “conversion disorder”, consist in the appearance of neurological symptoms that do not correspond to any medical condition and produces an impairment in social, occupational and other areas in the patient’s life. This disorder can represent up to 30% of neurologist’s consultation. We introduce the case of a 23-year-old man who attended the emergency services due to fainting and was finally diagnosed with FND.

**Objectives:**

To summarize the difficulties of making a diagnosis of FND and the importance of a multidisciplinary approach.

**Methods:**

A narrative review through the presentation of a case.

**Results:**

The patient presented many absence seizures during his stay in the hospital. These episodes were characterized by non-reactivity, dysarthria, tremors, tachycardia and hyperventilation. The neurological examination and imaging tests didn’t show any pathological findings. During the psychiatric interview he revealed he had lived a severe conflict with his brothers the previous week and he was being excluded within his family. Furthermore he didn’t have any social support besides his mother in the city he was living, leading this situation to an incrementation of anxiety. Due to the absence of any abnormalities in the examination and recent psychological conflict that was affecting him, FND diagnose was made.

**Conclusions:**

Very frequently the absence of a clear psychological trigger and the presence of neurological alterations can hinder the study of the patient. This makes necessary a multidisciplinary approach and the knowledge of signs that can help to carry out an accurate diagnosis.

**Disclosure:**

No significant relationships.

